# Supplementary material for: Are carbon emissions trading and green financial instruments synergistic? -Comprehensive quantitative research based on content analysis
Source: PLoS One. 2024 Mar 7;19(3):e0298601. doi: 10.1371/journal.pone.0298601 (PMC10919601; doi:10.1371/journal.pone.0298601)
Supplement: S1 Data — (ZIP) [file pone.0298601.s001.zip › Data/地级市Z_F工作报告.zip!/╔╜╢1⁄2/┴─│╟╩╨/└┤╫╘╣1⁄2╓┌║┼í╛┬φ┐╦╩2╛▌═°í┐_2014.docx]

各位代表：

　　现在，我代表市人民政府向大会报告工作，请予审议，并请市政协各位委员提出意见。

　　一、2013年工作回顾

　　刚刚过去的2013年，是全面贯彻党的十八大精神的开局之年，也是我市跨越赶超攻坚战首战告捷之年。一年来，在省委、省政府和市委的坚强领导下，全市上下紧紧围绕“东融西借、跨越赶超，建设冀鲁豫三省交界科学发展先行区”的奋斗目标，转轨提速、加压奋进，全市呈现出“经济健康发展、社会安定和谐、干部干事创业、人民安居乐业”的良好局面，各项目标任务全面完成。

　　（一）经济发展实现新跨越。按照跨越赶超的要求，全力推动科学发展，取得了“三个第一、两个提高、一个进位”的明显成效。预计全市生产总值完成2400亿元，比上年增长10%。公共财政预算收入完成135.6亿元，增长19.9%，增幅居全省第一位；国税收入完成119.4亿元，入地方库部分增长25.1%，增幅居全省第一位；地税收入完成93.4亿元，增长29%，增幅居全省第一位。公共财政预算收入占生产总值比重达到5.7%，比上年提高0.6个百分点;税收占财政收入比重达到74.5%，提高7.5个百分点。公共财政预算收入在全省前进了一个位次。各位代表，由于全市上下用心把握、用心推动、用心落实，三年跨越赶超第一战役取得了决定性胜利！

农业结构调整更加深入。粮食总产达到115.7亿斤，实现“十一”连丰。瓜菜菌总产达到1554万吨，居全省第一位，莘县被命名为“中国蔬菜第一县”。新增绿色食品认证119个，位居全省第一位。新增规模以上农业龙头企业50家，新增农民专业合作社2616个，农村基础设施建设三年会战全面启动，农业机械化率达到85%。工业加快转型升级。预计新增规模以上企业252家，主营业务收入达到8100亿元，增长17%，总量居全省第七位。大力开展全民创业、妇女创业、青年创领水城活动，民营经济蓬勃发展，私营企业和个体工商户增加2万户，增幅居全省第二位。新增国家级博士后工作站3家，东阿阿胶荣获首届中国质量奖提名奖，高新技术产业产值占规模以上工业比重提高了2个百分点，我市再次被评为全国科技进步先进市。节能减排工作进一步加强，我市代表山东省参加全国海河流域水污染防治检查，再次取得了第一名。服务业发展步伐加快。预计社会消费品零售总额增长13%以上，旅游总收入增长16.5%；服务业占生产总值比重达到34.7%，对地方财政贡献增长了37.3%。通过加快转方式调结构步伐，全市一二三产业不仅发展速度快，而且结构、质量、效益都有了明显提升！

　　（二）项目建设实现新突破。把加大投入作为跨越赶超的关键，狠抓招商引资和重点项目建设，预计固定资产投资达到1600亿元，增长21%。全市实际利用外资1.6亿美元，增长41.5%，增幅居全省第三位。招商引资到位资金720亿元，增长43%。新开工过亿元项目570个，比上年增加300个。年初确定的200个重点项目完成投资985亿元，比上年多完成182亿元，中通新能源客车、鲁西化工新材料、祥瑞铜材精密铜线、奥博特铜板带等79个项目建成投产。随着招商引资力度的不断加大，我市引进的大集团、大项目明显增多，与平安集团、光大集团、中建集团、中银国际、中科招商等知名企业建立了合作关系，全市在建过百亿元的项目5个、过50亿元的项目16个。各位代表，由于全市上下同心协力，用实招，求实效，我市全员招商、大上项目、上好项目的局面已经形成，为三年跨越赶超增强了支撑、增强了底气、增强了信心！

　　（三）改革开放实现新进展。坚持改革开放不动摇，努力为跨越赶超提供强大动力。各项改革加快推进。农村土地承包经营权确权颁证试点工作扩大到所有乡镇。鲁西、中通、鑫亚等股权多元化取得重大进展。食品药品和金融监管体制改革基本完成。新增股权挂牌企业21家，总数居全省第三位。大力推进市级以下财政体制改革，有力调动了县（市区）发展的积极性，全市公共财政预算收入过10亿元的县（市区）达到7个，比上年增加4个，东昌府区、茌平县超过20亿元；公共财政预算收入过1000万元的乡镇（街道）达到104个，比上年增加44个。这标志着“县域经济突破”战略取得了显著成效！“一区一圈一带”建设取得实质性进展。全年共争取各类资金157.6亿元，增长33.2%；有82个项目列入山东西部经济隆起带发展规划，占项目总数的1/5。与济南签署了战略合作框架协议，规划、交通、产业、要素、生态五个“一体化”加快推进。对外开放再上新的台阶。预计进出口总额完成62亿美元，首次跨入全国百强。“海外聊城”建设步伐加快，信发、祥光、冠丰、天工等境外资源开发、市场开拓取得明显成效。中通客车成功通过德国TüV标准认证，向德国出口新能源客车1000辆，标志着我国汽车首次进入德国市场。对外交流合作取得新进展，我市荣获美中友好协会“友谊了解奖”、“2013澳中友协国家奖”。 聊城经济技术开发区成功进入国家级开发区行列。

　　（四）城乡面貌实现新变化。大力实施“双百”大城市战略，全市住房和城乡建设完成投资921.6亿元，比上年增加了651.6亿元；预计城镇化率达到43%，提高3个百分点。城市发展框架进一步拉开。市城区形成了东昌府区、经济技术开发区、高新技术产业开发区、江北水城旅游度假区“四区”竞相发展的格局，环城道路、环城水系、环城林带、环城花带、环城高速“五环聊城”建设全面展开，各县（市）城区框架也明显拉大。城乡建设步伐进一步加快。全市棚户区改造征收面积完成1672万平方米，相当于过去10年的总和，居全省第一位。建成保障性住房12760套，完成率居全省第五位。改造农村危房15067户，居全省第一位。中心城区城建重点项目投资完成188.6亿元，比上年增加了86.6亿元，滨河大道全程通车，市体育场和高级工程、高级财经职业学校新校区投入使用，中华水上古城四条大街、市民文化中心等主体工程完工。城乡环境进一步改变。大力开展了城乡环境综合整治，全市新建改建道路238条，新增绿化面积705万平方米，林木绿化率达到34.6%，“平、明、绿、美、净、齐”的特点初步彰显。各位代表，过去的2013年，是我市城乡建设投入资金最多、改造力度最大、项目建设最快、面貌变化最明显的一年，也是广大人民群众最为理解支持、各级干部奉献付出最多的一年。通过全市上下的苦干实干，聊城的知名度和美誉度大幅提升，为推动“双百大城市”建设、加快新型城镇化发展奠定了良好的基础！

　　（五）要素支撑实现新增强。全市坚持攻坚克难，全力破解发展瓶颈。土地支撑更强。全市增减挂完成拆旧1.5万亩，复耕9169.8亩，是前5年的总和，居全省第一位；土地挖潜改造完成1.2万亩，建成标准厂房1504万平方米，全年报批土地4.9万亩，居全省第二位，有力地保障了项目建设！资金支撑更强。举办了三次大型金融合作洽谈活动，新引进外地银行3家，东昌府区农信社改制为聊城农村商业银行。截至去年年底，全市本外币存款余额达到1902亿元、贷款余额达到1430亿元，比年初分别增加214亿元和140亿元；表外业务余额达到515亿元，增长41%。交通支撑更强。邯济铁路扩能改造建成通车，德商高速全面开工，济聊一级公路征地拆迁工作已全面展开，青兰高速、莘县至南乐高速等前期工作基本完成，聊泰铁路、济聊城际铁路、牛角店至孝里连接线、德郓高速、军民合用机场、京九高铁、徒骇河通航等都取得重大进展，全市水陆空立体交通体系的框架正在拉开！

　　（六）发展环境实现新优化。坚持把优化环境作为推动发展的重大举措，加大了环境建设的力度。大力精简行政审批，市级审批事项由367项减少到96项，每年可为企业减负6000多万元，在全省率先做到了审批项目最少、速度最快、收费最低。大力提升服务水平，对投资过10亿元的项目，设立了警务室、派驻了联络员。大力开展“庸懒散”专项治理，建立健全了重大行政处罚备案制度和行政复议制度，“三乱”现象得到有效扼制，工作效率明显提高。大力优化社会环境，深入开展了“打黑除恶、打霸治痞”专项行动，所有乡镇建立了社会管理服务中心，人民来信来访、安全生产、防震减灾都取得了明显成效，社会保持了和谐稳定的良好局面。

　　（七）群众生活实现新改善。加快实施“民生优先”战略，财政用于民生的支出达到177.3亿元，占到总支出的68.7%，“八大民生实事”全部落实。全市城镇居民人均可支配收入达到26450元，农民人均纯收入达到10030元，分别增长11.5%和13%。我市卫生事业全省一流，是鲁西及周边地区市人民医院和市中医院唯一“双三甲”市，是全省唯一开展农村贫困家庭先心病儿童免费救治的市，县乡医疗机构全部实行了“先诊疗后付费”。我市教育教学改革全省一流，新建乡镇中心幼儿园135个，实现了乡镇（办事处）全覆盖；杜郎口中学、莘县实验小学教学改革走在全国前列，全市高考录取率位居全省上游。我市中心敬老院建设全省一流，“幸福敬老院”创建工作全面完成，新增社会养老床位5520张，全国农村五保供养服务机构建设推进会在我市召开。就业创业和社保水平明显提高，新增城镇就业6.2万人，新增农村劳动力转移就业7.1万人，城镇登记失业率为3.15%；城镇医疗保险、新农合补助标准每人每年提高到280元，大病医疗救助比例提高到55%，城市低保标准每人每年提高了300元以上，农村低保标准每人每年提高了400元，市儿童福利院全面建成、投入使用。文化体育等社会事业日趋繁荣，《萧城太后》等4个剧目荣获全国性大奖，高唐县成为海峡两岸书画交流基地；成功举办了全市第二届运动会，我市被授予全国群众体育工作先进单位；全面加强人口计生工作，人口自然增长率控制在7‰以内；国防动员、人民防空和民兵预备役建设进一步加强；广播电视、新闻出版、社会科学、档案、史志、气象、油区等工作都取得了新的成绩。

　　（八）自身建设实现新提升。坚持从严治政、严格管理，建立健全了政府规范运行的各项制度，组建了大督查落实委员会，加强对重点项目、重大工程、重要事项的督察，雷厉风行、狠抓落实之风已经形成。更加注重依法行政，人大代表建议、政协委员提案满意度达到98%。积极推进政务公开，市长公开电话群众满意率达到91%。严格执行“八项规定”，开展了纪律作风、厉行节约明察暗访，迎来送往、公款吃喝、铺张浪费现象明显减少，作风建设迈出实质性步伐。全面实行了公务卡制度，国库集中支付改革进一步深化，“三公”经费支出下降了10%。切实加强反腐倡廉建设，严肃查处了一批违法违纪案件，进一步提升了政府的良好形象。

　　各位代表，一年来，通过各级各方面的共同努力，全市各项工作都取得了显著的成绩，有13项指标排在了全省第一位。这些成绩的背后，是全市干部群众思想观念的重大转变、工作作风的重大转变、精神状态的重大转变。2013年，全市最大的变化，就是精神面貌为之一振，大家的注意力、兴奋点都聚焦到了发展上；最大的收获就是统一了思想、凝聚了共识，形成了同心同德、干事创业的浓厚氛围。一年来的实践充分证明，聊城要实现跨越赶超，必须用心谋划，把握准前进的方向。市委、市政府抓住“一区一圈一带”的重大机遇，作出了“东融西借、跨越赶超，建设冀鲁豫三省交界科学发展先行区”的总体部署，提出打好“三年翻番”、“进二争三”攻坚战，谋划了“富民兴聊八大战略”和“五大保障”，为全市发展指明了方向，对凝聚共识、引领发展起到了关键作用。必须解放思想，树立敢为人先的胆识。三次大规模的外出学习考察，使大家解放了思想、开阔了视野，增强了“别人能干，我们照样能干，而且能够干得更好”的斗志豪情，树立起逢一必争、逢旗必夺的胆识。必须加压奋进，激发干事创业的激情。市委、市政府像拧螺丝一样，对重点项目、三农工作、城镇化进行了四次大型观摩，并实名打分、当场公布。各级各部门压力大、干劲足，大家都比着干、争着干、拼着干，形成了百舸争流、竞相发展的生动局面。必须改革创新，增强攻坚克难的闯劲。在棚户区改造、土地增减挂、行政审批制度改革等难点问题上，各级都积极探索，大胆地试、大胆地闯，攻坚克难、破冰前行，不仅走开了路子，而且许多工作走在了全省的前列。必须领导带头，凝聚全市上下的合力。在招商引资、项目建设等各项重点工作中，各级领导亲力亲为、敢于担当，一级带着一级干、一级做给一级看，形成了攥紧拳、同心干的强大合力！

　　各位代表，一年来，全市经济社会发展形成这样的局面、取得这样的成绩，实属不易。这是省委、省政府和市委科学决策、坚强领导的结果，是市人大、市政协和社会各界加强监督、大力支持的结果，是全市上下同心协力、顽强拼搏的结果。在此，我代表市人民政府，向全市工人、农民、知识分子和企业家，向各民主党派、工商联、各人民团体和各界朋友，向驻聊人民解放军、武警官兵、公安干警和中央、省驻聊各单位，向所有关心、支持聊城发展的海内外朋友，表示衷心感谢！

　　各位代表，在看到成绩的同时，我们也清醒地认识到，我市在发展中还存在不少问题和不足。经济总量依然偏小，人均占有水平仍然较低；投资规模依然不足，投资结构还有待优化；产业结构依然不够合理，重化工业比重仍然偏高，多数产业处于价值链低端，服务业、高新技术产业有待提高；城镇化水平依然不高，与全省还有较大差距；节能减排压力依然较大，大气污染治理形势仍很严峻；社会保障体系依然不够完善，社会事业还难以满足群众的实际需要；政府职能转变依然不够到位，经济发展环境还有待进一步优化。对这些问题，我们一定要高度重视，下决心予以解决。

　　二、 2014年的目标任务

　　各位代表，今年是全面贯彻党的十八届三中全会精神、全面深化改革的第一年，是圆满完成“十二五”规划任务目标的关键一年，也是打好三年跨越赶超攻坚战至关重要的一年。今年工作的总体要求是：认真贯彻党的十八大、十八届三中全会和习近平总书记一系列重要讲话精神，按照市委十二届四次、五次全会的部署要求，坚持稳中求进、改革创新，着力打好重点项目建设、产业转型升级、深化改革开放、加快城镇化、保障和改善民生五场硬仗，确保综合实力明显增强、发展质量明显提升、城乡面貌明显变化、生态环境明显改善、社会事业明显进步、群众生活明显提高、机关作风明显改进，坚决打好东融西借、跨越赶超攻坚战第二战役，为建设冀鲁豫三省交界科学发展先行区奠定坚实基础。主要预期目标是：生产总值增长10%，公共财政预算收入增长15%，固定资产投资增长21%，社会消费品零售总额增长13.5%，实际利用外资增长10%，进出口总额增长11%，城镇居民人均可支配收入增长12%，农民人均纯收入增长13%，居民消费价格涨幅控制在3.5%以内，城镇登记失业率控制在3.7%以内，节能减排、人口自然增长率等全面完成省下达的任务。

　　各位代表，今年预期目标的确定，主要考虑要与我市“十二五”规划相衔接、与三年跨越赶超的目标相衔接，同时为转方式调结构留出空间。在实际工作中，我们仍要坚持自抬标杆、加压奋进，使科学发展、跨越发展成为全市的最强音，努力使发展速度高于全省平均水平、高于周边市水平、高于去年的水平，在跨越赶超进程中迈出更大更坚实的步伐！

　　（一）着力在转调上狠下功夫，确保综合实力更强。坚持科学发展，加大转方式调结构力度，推动经济发展的速度、质量、效益全面提升。

　　加快发展现代农业。大力实施“农业龙头带动”战略，着力在农业“高产化、产业化、规模化、品牌化”上实现突破。一是加快农业“高产化”。继续打好农村基础设施建设三年会战，加快南水北调平原水库、大型引黄灌区节水、中小河流治理、小农水重点县等工程建设。抓好全国新增千亿斤粮食产能、省千亿斤粮食产能规划项目建设，落实高产示范田100万亩，大力提升机械化水平，加快建设“吨粮市”，为国家粮食安全做出贡献。二是加快农业“产业化”。加大农业龙头企业培育力度，新增规模以上龙头企业80家，龙头企业产值突破1000亿元。加快聊城农产品物流交易中心建设，重点培育10处年交易额2亿元以上的农产品批发市场。大力推进农科对接、农金对接、农超对接，抓好邮政万亩示范田建设，推广供销系统“大田托管服务”，尽快构建起完善的农业产业体系。三是加快农业“规模化”。抓好100家市级农民专业合作社建设，力争辐射带动农户率达到40%。大力培育家庭农场、专业大户、农业企业等经营主体，土地流转面积达到120万亩。四是加快农业“品牌化”。集中力量培育莘县香瓜、冠县鸭梨等12个名优特色产品，打造区域特色品牌。以甲鱼、湖羊、锦鲤等为重点，打造全国最大的精致农业生产基地。抓好市级农产品综合质检中心建设，确保新增“三品一标”品种100个、基地面积40万亩，打造全国最大的绿色安全蔬菜生产基地。各位代表，聊城作为农业大市，必须把“三农”工作摆在重中之重的位置，在现代农业发展上走在全省前列，努力让农业成为有奔头的产业，让农民成为体面的职业，让农村成为安居乐业的美丽家园！

　　推动工业优化升级。深入实施“万亿工业”战略，以企业“二次创业”为抓手，强化创新驱动，力争规模以上工业主营业务收入过万亿元。一是壮大一批。围绕培植战略支柱产业和优势特色产业，大力推进“3+5”千亿产业园建设，着力抓好产业链拉长、价值链高端、科技资源集聚，打造国际国内具有竞争力的产业集群，完成投资350亿元，主营业务收入达到3400亿元。二是提升一批。围绕传统优势产业，支持企业加大研发和技改投入，提高精深加工产品、高附加值产品的比重，全年技术改造投资增长17%以上。三是扶持一批。实施“2150”企业培育工程，用3年时间培植主营业务收入过千亿元的企业2个，过100亿元的企业10个，过50亿元的企业50个。四是培育一批。加强与聊城大学的合作，建设鲁西大学科技城，积极引进高端人才，建设人才高地。加强企业创新平台建设，支持企业开展产学研合作。抓好中通客车、高唐时风、莘县巨龙等新能源汽车的发展，加快山东抗体制药协同创新中心、中原生物医学研究院等七个研发机构建设，努力打造全国最大的新能源汽车、全省最大的生物制药产业基地，高新技术产业产值占规模以上工业比重提高2个百分点以上。

　　加快推进服务业跨越发展。深入实施“服务业倍增战略”，确保服务业增加值达到1000亿元，所占比重提高2个百分点。一是大力发展文化旅游业。突出水城特色，抓好黄河天音旅游度假区、马颊林海度假区、东昌古郡游憩区、凤凰绿岛养生休闲区四大旅游综合体建设。大力推进江北水城旅游度假区建设，打造高端旅游发展核心区。整合温泉休闲疗养、阿胶养生养颜、梵呗音乐养心等资源，打造“中国养生首选地”。加快发展乡村旅游，打造一批特色名牌。突出抓好中华水上古城、运河文化大码头、江北水镇等重大项目建设，尽快形成一批具有吸引力、震撼力的旅游景区。抓好南关岛、北关街等特色商贸街区建设，丰富文化旅游元素。二是加快发展商贸物流业。抓好聊城物流园、汇通金属商贸物流园等10大物流园区建设改造，加快推进盖世物流、天龙物流等10家重点企业发展，支持聊城鑫鹏集团网上钢材交易平台等一批电子商务企业发展，全面提升商贸物流业的规模和水平。三是积极发展其他服务业。鼓励发展金融、信息、研发、商务服务、教育培训、医疗健康、社区服务等现代服务业，积极培育信息软件、动漫创意、网络媒体等新兴服务业，促进全市服务业转型升级。各位代表，在推动聊城跨越发展的进程中，我们一定要强化科学发展的理念，追求实实在在、没有水分的速度，追求发展活力更强、质量效益同步提高又不带来后遗症的速度，加快我市产业结构由目前的“二三一”向“三二一”的根本性转变！

　　（二）着力在项目建设上狠下功夫，确保发展后劲更足。坚持“大发展要有好项目、快发展要有大项目”，着力优化投资结构，固定资产投资力争突破2000亿元。继续实行全员招商，进一步调动各级各部门的积极性，努力在总量上实现突破，确保完成招商引资840亿元以上。突出招大引强，瞄准世界500强、国内500强和行业50强，积极主动搞好对接，努力在大项目引进上实现突破。突出产业链招商、产业集群招商、高新技术产业招商，努力在价值链高端、高科技、高附加值项目引进上实现突破。完善重大招商项目落地联席会议制度，挖潜改造土地1万亩，土地增减挂完成验收1万亩，努力在项目落地上实现突破。加强专业招商队伍建设，深化与各地商会和中介机构的沟通合作，努力在招商体系建设上实现突破。抓好项目谋划和争取工作，充实调整“212”项目库，努力在投资过百亿元项目、列入国家和省重点项目数量上实现突破。集中力量抓好投资236亿元的信发集团铝电升级改造、106亿元的泉林秸秆综合利用等200个重点项目建设，努力在重点项目建设上实现突破，全年完成投资1160亿元。打好三年跨越超越攻坚战，项目建设是关键。我们必须坚持不懈地狠抓招商引资、狠抓项目建设，着力培植一批新的产业支柱，积蓄起跨越赶超的强大后劲！

　　（三）着力在放开搞活上狠下功夫，确保民营经济发展更快。民营经济是我市的短板，也是潜力所在，必须加大扶持支持力度。一是加快规模扩张。大力实施“11236”工程，深入推进全民创业，加快小企业创业辅导基地建设，新建标准化厂房200万平方米以上，确保个体工商户和私营企业户数新增3万户以上。二是加快质量提升。组织开展好“一企一技术”、管理提升“百千万”、中小微企业创新服务等活动，积极引导企业采用新技术、新工艺、新装备，支持企业创建国家和省级名牌，确保民营经济税收增长26%以上。三是加快集群发展。围绕轴承、板材、畜禽、钢管、钎具等五大民营特色产业，抓好30个特色产业镇、500个特色产业村发展，打造一批特色鲜明、行业领先的产业集群。各位代表，民营经济是实现跨越赶超的重要力量。各级一定要放手、放胆、放开，努力让一切创造社会财富的源泉充分涌流、让干事创业的活力竞相迸发，以民营经济的快速发展促进全市的跨越赶超！

　　（四）着力在生态宜居上狠下功夫，确保城乡面貌更新。加快实施“双百大城市”战略，努力打造全国最大的生态水城，全市城镇化率提高2个百分点以上。一是拉大城市框架。坚持高水平规划，完成城市总体规划修编和聊茌东大三角规划编制。全面加快“四区”建设，到2015年，市经济技术开发区跨入国家级开发区百强，高新技术开发区跨入国家级行列，江北水城旅游度假区要建成省级知名度假区。抓好东环路以东“三纵四横”和湖南路以南“三纵三横”道路建设改造，加快推进东扩南展。高标准建设“五环聊城”，确保城市环境明显提升。各县（市区）也要以新城区、经济技术开发区、工业园区和交通干线沿线为重点，进一步拉开城市框架、扩大城市规模。二是提升建设水平。着力完善城市功能，抓好市政设施、文体设施和绿地公园建设，中心城区新增绿地面积220万平方米。抓好20个城建重点项目建设，确保市民活动中心等8个项目全面完成。坚持高标准、高起点，集中力量打好回迁安置房建设攻坚战，确保按时回迁、取信于民。三是突破小城镇建设。突出抓好22个省、市示范镇建设，全面完成规划编制，加快基础设施建设，不断提高综合服务功能。坚持小城镇建设与产业发展一体推进，加快形成一批商贸带动型、工业带动型、旅游带动型和交通带动型的特色小城镇。四是提升城乡管理水平。在农村，实施生态文明乡村建设“百千万工程”。用三年时间，每年建设100个示范村（社区），对1000个村（社区）进行环境综合整治，让100万农村居民受益，加快建设美丽乡村。在城区，按照“平、明、绿、美、净、齐”的标准，大力开展城市环境综合治理，进一步提升城市形象。各位代表，城镇化事关现代化，事关子孙后代，必须坚持长远眼光，必须坚持天人合一，科学规划，务实行动，真正把聊城建设成为宜居、宜业、宜游的美丽家园！

　　（五）着力在深化改革上狠下功夫，确保体制机制更活。坚持以改革促发展，扎实有序地推进各项改革。一是深化农村改革。全面完成农村集体土地所有权、宅基地使用权和建设用地使用权确权登记发证，农村土地承包经营权确权颁证完成50%以上。二是建立完善现代企业制度。积极推动国有企业对外合资合作，鼓励国有资本、集体资本和非公有资本交叉持股，发展混合所有制经济。三是扎实推进金融改革。加快金融集聚区规划建设，积极引进各类金融机构。大力推进企业上市和新三板挂牌，力争突破境外上市。支持农村信用社银行化改革，引导符合条件的大型企业集团组建财务公司，鼓励发展小额贷款公司和担保机构，积极稳妥地发展民间资本管理服务机构。四是深化财税改革。深入推进财政体制改革，做好“营改增”工作。大力整合各类基金，集中力量扶持重点领域发展。加快构建全面规范、公开透明的预算制度，对财政拨款过1000万元的投资发展类项目，全部实行绩效管理。加强政府债务管理，切实防范债务风险。五是推进城乡一体化改革。全面放开建制镇和小城镇落户限制，降低中心城区落户门槛，推进城乡人口一体化管理，促进农村居民加快向城镇转移，让更多的农民群众过上与城里人一样的生活！

　　（六）着力在对外开放上狠下功夫，确保发展空间更广。坚持把开放型经济发展放在重要位置，切实利用好国际国内两种资源、两个市场。一是区域合作要有新进展。更加积极主动地融入省会城市群经济圈，着力在五个“一体化”上取得新进展，努力把“聊茌东”大三角建成融入“一区一圈一带”的产业发展新高地。二是对外贸易要上新水平。鼓励出口企业调整商品结构，打造出口品牌。积极引导企业参与国际贸易，争取进出口获权企业达到1000家。加强区域合作，积极参与构建对外经济走廊。加快聊城保税物流中心、冠县宝信物流、阳谷凤祥保税仓库和山东-京畿道（韩国）产业合作园建设，推进对外贸易便利化。三是“海外聊城”建设要有新成效。大力实施走出去战略，积极稳妥地推进境外工业园区建设，支持中通客车、昌润集团等企业加大境外并购扩张力度，更好地开拓国际市场。整合鲁西国际公司等外派劳务培训机构，打造鲁西人力资源品牌。四是开发区发展要上新台阶。要进一步加强基础设施建设，加大招商力度，加快发展步伐，力争所有省级经济技术开发区在全省综合排名提高3个位次。各位代表，聊城对内对外的开放都面临着难得的重大机遇，我们必须实施更加积极主动的开放战略，下好先手棋，打好主动仗，让聊城的开放度更高、竞争力更强，让聊城发展的航船驶向更广阔的海洋！

　　（七）着力在交通建设上狠下功夫，确保基础支撑更牢。要抓住用好“一区一圈一带”重大机遇，大力实施“立体大交通”战略。一是抓好重大交通设施建设。加快德商高速聊城段建设，确保2015年建成通车。力争济聊城际铁路、聊泰铁路、青兰高速、莘县至南乐高速、济聊一级公路、牛角店至孝里连接线开工建设。确保军民合用机场、德郓高速、聊城至黄骅港铁路、京九高速铁路、聊城至石家庄铁路、徒骇河通航等重大项目前期工作取得新的进展。二是抓好干线和农村公路建设。对市与县（市）之间、县与县（市）之间干线公路，按照特一级公路标准改造升级，重点抓好聊城到东阿、聊城到夏津等四条道路建设，新建、改建干线公路140公里。加强农村公路建设维护，新建改建农村公路1000公里。三是抓好路域环境综合整治。以绿化、美化、净化为重点，对高速路、国省道、环城路及道路结合部进行综合整治，实现道路整洁、三季有花、四季常绿，打造“畅安舒美”的路域环境。各位代表，交通是发展的重要支撑。随着这些重大交通项目的建成，聊城将形成高速成网、高铁叠加、飞机起降、轮船出海的水陆空立体大交通体系，真正成为重要的交通枢纽城市！

　　（八）着力在节能减排上狠下功夫，确保环境质量更优。牢固树立既要金山银山、更要碧水蓝天的理念，大力实施“生态水城”战略。一是狠抓能源节约。突出抓好重点企业节能工作，实施节能工程14项。大力发展循环经济，将聊城经济技术开发区、高唐工业园建设成为国家级示范园区。在全社会推进能源节约，实施建筑节能改造40万平方米、太阳能光热建筑一体化75万平方米。二是狠抓大气污染防治。大力调整能源结构，严格控制新建燃煤电厂，鼓励使用天然气等清洁能源，建设国家级新能源汽车示范市。实行最严格的节能减排标准，所有燃煤企业必须按新标准进行脱硫除尘升级、建设脱硝工程。加强源头治理，推行扬尘网格化管理，抓好企业异味等面源废气治理，实施鲁西集团三个分厂退城进园。严格环保执法，对违规企业从重从严处罚。三是狠抓生态保护。加强雨洪资源收集利用，实施地下水超采漏斗区综合治理。积极推进农村面源污染治理，推广秸秆综合利用和新型能源。狠抓植树造林，林木绿化率达到37.5％以上。加快水污染防治重点工程建设，抓好23块人工湿地改造提升，彻底解决污水直排问题。各位代表，聊城是我们共同生活的家园，让我们携起手来、一起努力，确保让全市人民喝上放心水、呼吸上新鲜空气！

　　（九）着力在保障民生上狠下功夫，确保群众生活更好。坚持以人为本、执政为民，大力实施“民生优先”战略，推进社会事业全面进步。一是优先发展教育事业。全面抓好学前教育、义务教育和高中阶段教育，鼓励发展民办教育，促进教育均衡发展，便利城乡居民子女就近入学。发挥好聊城大学、聊城职业技术学院、山东工程技师学院、聊城技师学院、东昌学院等教育资源优势，打造冀鲁豫三省交界地区教育和人才培养高地。二是积极发展卫生事业。充分发挥国有医疗资源优势，加快建设省级区域医疗中心。抓好县级公立医院改革试点，推动基本药物制度向城市社区卫生服务机构延伸。坚持计划生育基本国策，稳定低生育水平。三是繁荣发展文化体育事业。实施道德建设“四德工程”，扎实开展文明城市、文明村镇、文明行业等创建活动。市民文化中心要全面建成、投入使用，启动市图书馆建设。要切实做好广播电视、新闻出版、社会科学、档案、史志等工作，丰富人民群众精神文化生活。加强公共体育设施建设，广泛开展全民健身运动。四是全面抓好“平安聊城”建设。强化安全生产“红线”意识，突出企业主体责任，强化对重点行业和重点领域的监管，加强防震减灾、气象和应急管理，抓好输油输气管网管护，坚决遏制重特大安全事故发生，确保人民群众生命财产安全。认真贯彻党的民族宗教政策，扎实做好外事侨务、对台交流、来信来访工作。进一步加强国防动员、人民防空和民兵预备役工作，促进军地、军民团结。深入开展普法教育，努力创建“全国法治城市”。加强社会治安综合治理，完善社会治安防控体系，确保社会和谐稳定！

　　各位代表，在推动各项社会事业健康发展的同时，要坚持求真务实，集中力量办好“十大民生实事”。(1)加强就业扶持。设立市级创业带动就业扶持资金，加大小额担保贷款支持力度，做好以高校毕业生为重点的青年就业、农村劳动力转移就业和城镇困难群体就业工作，全市城镇新增就业4.5万人，新增农村劳动力转移就业5万人。(2)提高保障水平。整合城乡居民基本医疗保险，提高政府补助标准，增加大病保险种类，提高大病救助水平。进一步提高城乡低保标准，抓好进城落户农民、被征地农民社会保障工作。推进农村五保规范化管理，新增养老床位4000张。强化城乡社会救助，解决特困家庭生活问题。(3)改善办学条件。启动第二轮学前教育三年行动计划，建设市直实验幼儿园，新建改造农村校舍35万平方米，将聊城幼儿师范升格为高等专科学校。深入开展校园周边环境整治，确保师生安全。(4)强化医疗服务。开展“百名专家援基层、千名主治进乡村”行动，努力让基层群众享受到优质的医疗服务。继续开展农村贫困家庭先心病儿童免费手术，为2700名贫困白内障患者免费实施复明手术。(5)实施文化惠民“三千”工程。举办文艺演出下基层1000场，为全市1000个农家书屋、1000个村文化大院配置文化设施。（6）大力发展公共交通。实施公交优先战略，中心城区更换节能环保公交车100部，新开辟公交线路5条，延长运营时间，增加车辆班次，落实专用车道，使公共交通更加快速便捷。（7）完善生活设施。中心城区新建集中供热管网33公里，集中供热普及率达到70%以上；新增天然气管网35公里，新建小区全部通上天然气。（8）加强住房建设。建设棚户区回迁安置房6.6万套、保障性住房3.8万套，实施农村危房改造1.6万户。（9）抓好扶贫开发。深入开展百村万户脱贫工程，支持100个村发展致富产业，确保贫困户收入大幅增加。（10）保障食品药品安全。完善食品药品安全监管机构，加强监管能力、检验检测能力和信息化建设，全面建成初级农产品质量可追溯体系，确保人民饮食用药安全，争创食品安全放心市。各位代表，民生大于天,枝叶总关情。各级要通过扎扎实实的工作，进一步织牢社会保障网，努力让全市人民生活得更殷实、更体面、更有尊严！

　　三、加强政府自身建设

　　打铁还须自身硬。今年改革发展的艰巨任务，对政府自身建设提出了更高的要求。我们必须坚持改革创新，着力转职能、改作风，加快建设高效、责任、务实、法治、廉洁的人民政府。

　　（一）切实转变职能，建设高效政府。转变职能、提高效率是民之所望、施政所向。要按照十八届三中全会要求，大力推进改革创新，推动政府工作全面提速。要继续清理精减审批事项，保持在全省审批项目最少、速度最快、收费最低。要加强电子政务建设，推行网上审批、网上办公。要下大力精简会议和文件，开短会、发短文、讲短话，努力提高行政效率。要严格要求、严格管理，切实做到令行禁止、政令畅通，日事日毕、日清日结，确保政府服务更优、节奏更快、效率更高！

　　（二）切实为民服务，建设责任政府。立党为公，人民至上。我们要始终坚持把人民群众放在心中最高位置，把群众满意作为第一追求，切实维护好、发展好、实现好全市广大人民群众的根本利益。要坚持问政于民、问计于民、问需于民，做好人民建议征集工作，政府重大决策特别是关系群众切身利益的决策，要广泛征求群众的意见。要进一步做好来信来访、市长公开电话工作，建立网上受理平台，及时处理、认真整改群众反映的问题。要加强与群众的联系，经常深入问题多、困难大、矛盾集中的基层单位，倾听群众呼声、帮助群众解决实际问题，扎扎实实为群众办实事、办好事，真正担负起“兴一方经济、富一方百姓、建一方文明、保一方稳定”的政治责任。

　　（三）切实改进作风，建设务实政府。一分部署、九分落实，行动最具说服力。我们要以踏石留印、抓铁有痕的狠劲抓落实，说了算、定了干，言必信、行必果，确保“定一件、干一件，干一件、成一件”。要建立完善目标管理责任制，把经济社会发展目标逐级分解，做到项目化、数量化、具体化，做到时间倒推、工期倒排、到人到时到标准。继续抓好“庸懒散”整治，坚决制止“三乱”行为。进一步抓好大督察工作，加强对重点项目、重大工程、重要事项的督察，确保把各项工作落到实处、取得实效。

　　（四）切实依法行政，建设法治政府。法治严明事业兴，政府治理和政府建设必须具有合法性和有效性。要坚持依法民主科学决策，健全重大事项决策制度，抓好风险评估和合法性审查，加强跟踪反馈和责任追究。大力推进行政执法规范化，认真清理基层执法项目，规范行政处罚裁量权，推进跨部门、跨行业的综合执法。要讲究工作方法，依法和谐做好土地征用、房屋征收等重点工作。进一步完善行政复议工作，健全政府法律顾问制度。自觉接受人大及其常委会依法监督和政协民主监督，落实好人大及其常委会的决定决议，认真办理好人大代表建议和政协委员提案。加强司法监督和监察、审计等专项监督，主动接受新闻舆论和群众的监督。深入推进政务公开，进一步扩大公开范围，拓宽公开渠道，确保政府权力在阳光下运行。

　　（五）切实反腐倡廉，建设廉洁政府。为政之本，在于清廉。各级政府要坚持一要干事，二要干净，扎实落实好“一岗双责”。推行政府及工作部门权力清单制度，依法公开权力运作流程。加快建立全市统一的资源交易市场，完善落实国库集中支付制度、公务卡制度和收支两条线规定，推进“三公”经费和财政预算公开，切实堵住各种漏洞，把权力装进制度的笼子。严格执行中央、省委、市委出台的各项规定，厉行勤俭节约，狠刹奢侈浪费之风，确保各级政府非生产性支出下降20%以上。严肃查处违纪违法案件，努力做到干部清正、政府清廉、政治清明，进一步树立政府的良好形象！

　　各位代表，改革创新风正劲，跨越赶超战鼓催。让我们更加紧密地团结在以习近平同志为总书记的党中央周围，在省委、省政府和市委的坚强领导下，同心同德，务实求进，奋战2014、打赢第二战役，为建设冀鲁豫三省交界科学发展先行区、创造聊城人民更加美好的幸福生活做出新的更大的贡献！
